# DNA methylation of the allergy regulatory gene interferon gamma varies by age, sex, and tissue type in asthmatics

**DOI:** 10.1186/1868-7083-6-9

**Published:** 2014-05-22

**Authors:** Stephanie Lovinsky-Desir, Robert Ridder, David Torrone, Christina Maher, Surinder Narula, Melissa Scheuerman, David Merle, Meyer Kattan, Emily DiMango, Rachel L Miller

**Affiliations:** 1 Division of Pediatric Pulmonology, Department of Pediatrics, College of Physicians and Surgeons, Columbia University, 3959 Broadway CHC-737, New York, NY 10032, USA; 2 Division of Pulmonary, Allergy and Critical Care of Medicine, Department of Medicine, College of Physicians and Surgeons, Columbia University, PH8E-101, 630 W 168 St, New York, NY 10032, USA; 3 Department of Epidemiology, Mailman School of Public Health, Columbia University, 722 W 168 St, New York, NY 10032, USA; 4 Department of Environmental Health Sciences, Mailman School of Public Health, Columbia University, 722 W 168 St, New York, NY 10032, USA; 5 Division of Pediatric Allergy, Immunology and Rheumatology, Department of Pediatrics, College of Physicians and Surgeons, Columbia University, PH8E-101, 630 W 168 St, New York, New York 10032, USA

**Keywords:** Interferon gamma, Methylation, Buccal cells, CD4+ lymphocytes, Age-related methylation, Sex-related methylation, Tissue specific methylation, Asthma, Epigenetics

## Abstract

**Background:**

Asthma is associated with allergic sensitization in about half of all cases, and asthma phenotypes can vary by age and sex. DNA methylation in the promoter of the allergy regulatory gene interferon gamma (IFNγ) has been linked to the maintenance of allergic immune function in human cell and mouse models. We hypothesized that IFNγ promoter methylation at two well-studied, key cytosine phosphate guanine (CpG) sites (-186 and -54), may differ by age, sex, and airway versus systemic tissue in a cohort of 74 allergic asthmatics.

**Results:**

After sampling buccal cells, a surrogate for airway epithelial cells, and CD4+ lymphocytes, we found that CD4+ lymphocyte methylation was significantly higher in children compared to adults at both CpG sites (*P* <0.01). Buccal cell methylation was significantly higher in children at CpG -186 (*P* = 0.03) but not CpG -54 (*P* = 0.66). Methylation was higher in males compared to females at both CpG sites in CD4+ lymphocytes (-186: *P* <0.01, -54: *P* = 0.02) but not buccal cells (-186: *P* = 0.14, -54: *P* = 0.60). In addition, methylation was lower in CD4+ lymphocytes compared to buccal cells (*P* <0.01) and neighboring CpG sites were strongly correlated in CD4+ lymphocytes (r = 0.84, *P* <0.01) and weakly correlated in buccal cells (r = 0.24, *P* = 0.04). At CpG -186, there was significant correlation between CD4+ lymphocytes and buccal cells (r = 0.24, *P* = 0.04) but not at CpG -54 (r = -0.03, *P* = 0.78).

**Conclusions:**

These findings highlight significant age, sex, and tissue-related differences in IFNγ promoter methylation that further our understanding of methylation in the allergic asthma pathway and in the application of biomarkers in clinical research.

## Background

Asthma is an environmentally triggered disease that is linked to allergic sensitization in approximately 40 to 60% of cases [1]. Critical to the development of allergic asthma is the differentiation and maintenance of T helper (Th) cells with a Th2 instead of a Th1 cytokine profile and their suppression by the action of T regulatory (Treg) cells [2-4]. The Th1 cytokine interferon gamma (IFNγ) is key to this counter-regulation and has been shown to be epigenetically regulated. For example*, in vivo* IFNγ gene expression is reduced in CD4+ T cells of asthmatics versus non-asthmatics [5]. Several studies to date have demonstrated altered methylation in the promoter region of the IFNγ gene as a mechanism for silencing or inducing Th1 polarization and/or differentiation [6-11] in both murine (cytosine phosphate guanine (CpG) -34, -45, -53) [12] and human (CpGs -53, -186) cells [13].

Emerging evidence suggests that environmental triggers for allergic asthma can alter DNA methylation in the IFNγ gene promoter and thereby modify asthma risk [14,15]. Two recent murine studies demonstrated that fungal allergen [14] and ovalbumin (OVA) [15] sensitization, models of allergic disease, increased IFNγ promoter methylation in splenic CD4+ T lymphocytes. Hypermethylation of IFNγ was positively correlated with proallergic immunoglobulin E (IgE) [14] and associated with protection from allergic inflammation and reduction in airway hyperreactivity in experimental versus control mice [15]. This relationship was investigated further both *in vitro* and in human cell experiments by Tang *et al.*[16]. Jurkat T cells were exposed to the environmental air pollutant polycyclic aromatic hydrocarbon (PAH) and demonstrated enhanced IFNγ promoter DNA methylation and diminished gene expression. Furthermore, in human umbilical cord white blood cells, IFNγ promoter methylation was higher in children with higher measures of prenatal PAH exposure compared to lower ones, measured by personal monitoring for 48 hours during pregnancy [16]. IFNγ methylation also has been shown to vary by asthma diagnosis among monozygotic twins consistent with mediation by environmental exposure [5].

DNA methylation is dynamic [17-19]. Though global methylation appears to increase over time [20], studies by both Melvin *et al.* and White *et al.* demonstrated that IFNγ T cell methylation appears to decrease in adults compared to neonates [21,22]. These relationships, however, have not been previously explored in a population of allergic asthmatics. Similarly, DNA methylation has been shown to vary by sex [23,24], though these differences have not been well-characterized, particularly those relating to asthma regulatory genes. Moreover, asthma is characterized by a male predominance in childhood and female predominance in adulthood [25,26], a relationship that could be linked to age and sex-related variation in DNA methylation.

Finally, differences in gene expression across tissues have been shown to be important in asthma. For example, in a small cohort of 25 children with and without atopic asthma, Stefanowicz *et al.*[27] demonstrated 80 CpG sites across 67 genes that were differentially methylated in peripheral blood mononuclear cells (PBMCs) compared with airway epithelial cells (AECs) [27], suggesting each cell type has its own unique methylation signature. Similarly, in a newborn twin cohort in which cord blood mononuclear cells and granulocytes, human umbilical vein endothelial cells, buccal cells, and placental tissue was obtained, varying patterns of methylation were noted between tissue types in the insulin growth factor 2 (IGF2) and H19 gene loci [28]. Both systemic and target tissue-specific biomarkers are utilized in asthma research, thus a better understanding of methylation differences in varying cell types is critical as we explore appropriate biomarkers for epigenetic asthma research.

**Figure 1:**
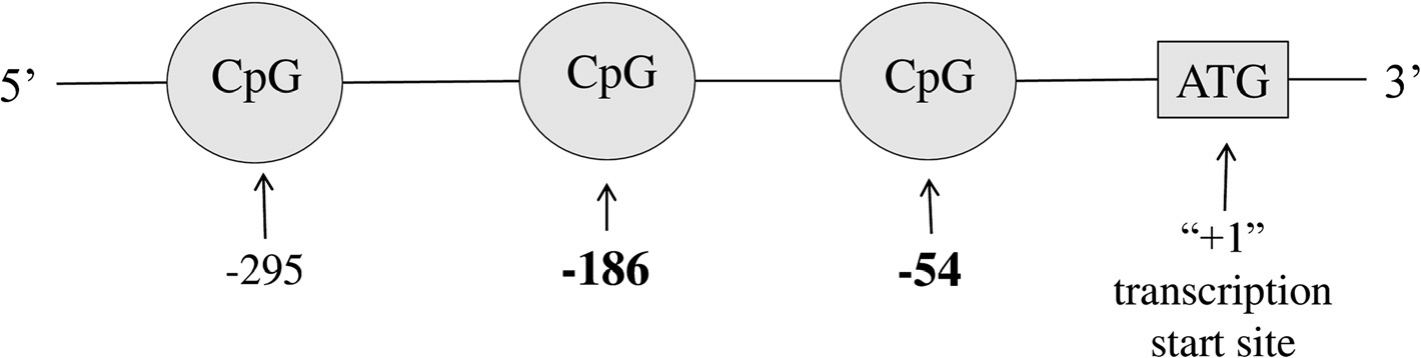
**Schematic diagram of the location of CpG sites targeted in the promoter region of the IFNγ gene.** CpG -54 corresponds to the murine counterpart CpG -53. Methylation levels at CpGs -54 and -186 have been shown to be mediated by environmental exposures and in association with allergic outcomes in animal models [14,15] and asthma in human studies [5,31]. CpG -295 is not conserved between mice and humans.

We hypothesized age and sex-related differences exist in DNA methylation of the allergy counter-regulatory gene IFNγ in a cohort of children and adults with allergic asthma. We also hypothesized that methylation in systemic CD4+ T lymphocytes differs from buccal cells, a lower airway epithelial cell surrogate. We sought a biomarker that may be suitable for wide application in pediatric clinical research and be much less invasive than the sampling of lower airway epithelial cells, the disease target cells. Buccal cells, an easily accessible population of neighboring tissue that encompasses a portion of the upper airway, have been previously proposed as a surrogate [29,30]. In this current study we sought to compare DNA methylation levels at highly conserved (between human and mouse) CpG sites in the IFNγ gene promoter region (CpG -186 and -54, Figure 1) between CD4+ T lymphocytes and buccal cells in a select cohort of allergic-sensitized children and adults with asthma. CpGs -186 and -54 also were selected because of their association with environmental exposure and allergic outcomes in animal studies [14,15], as well as with asthma outcomes in human studies [5,31].

## Results

### Variation in methylation levels by age and sex

**Figure 2:**
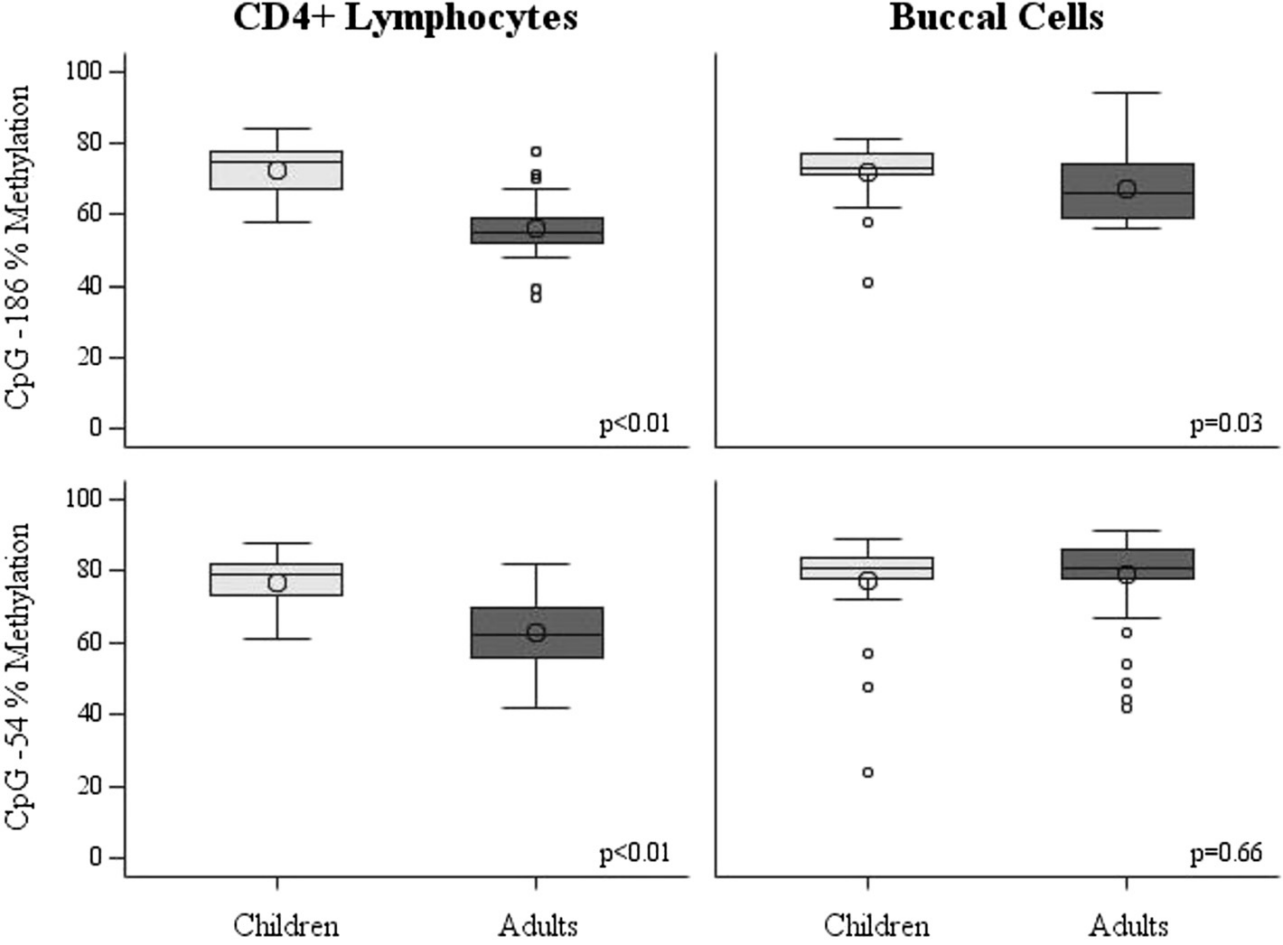
**Comparison of IFNγ promoter methylation levels by tissue type and CpG site stratified by age.** Children <18 years, n = 27, adults ≥18 years, n = 47. Children had significantly higher CD4+ methylation levels at both CpG sites (-186 and -54: *P* <0.01). The methylation level of buccal cells was also significantly higher in children compared with adults at CpG -186 (*P* = 0.03) but not CpG -54 (*P* = 0.66).

**Figure 3:**
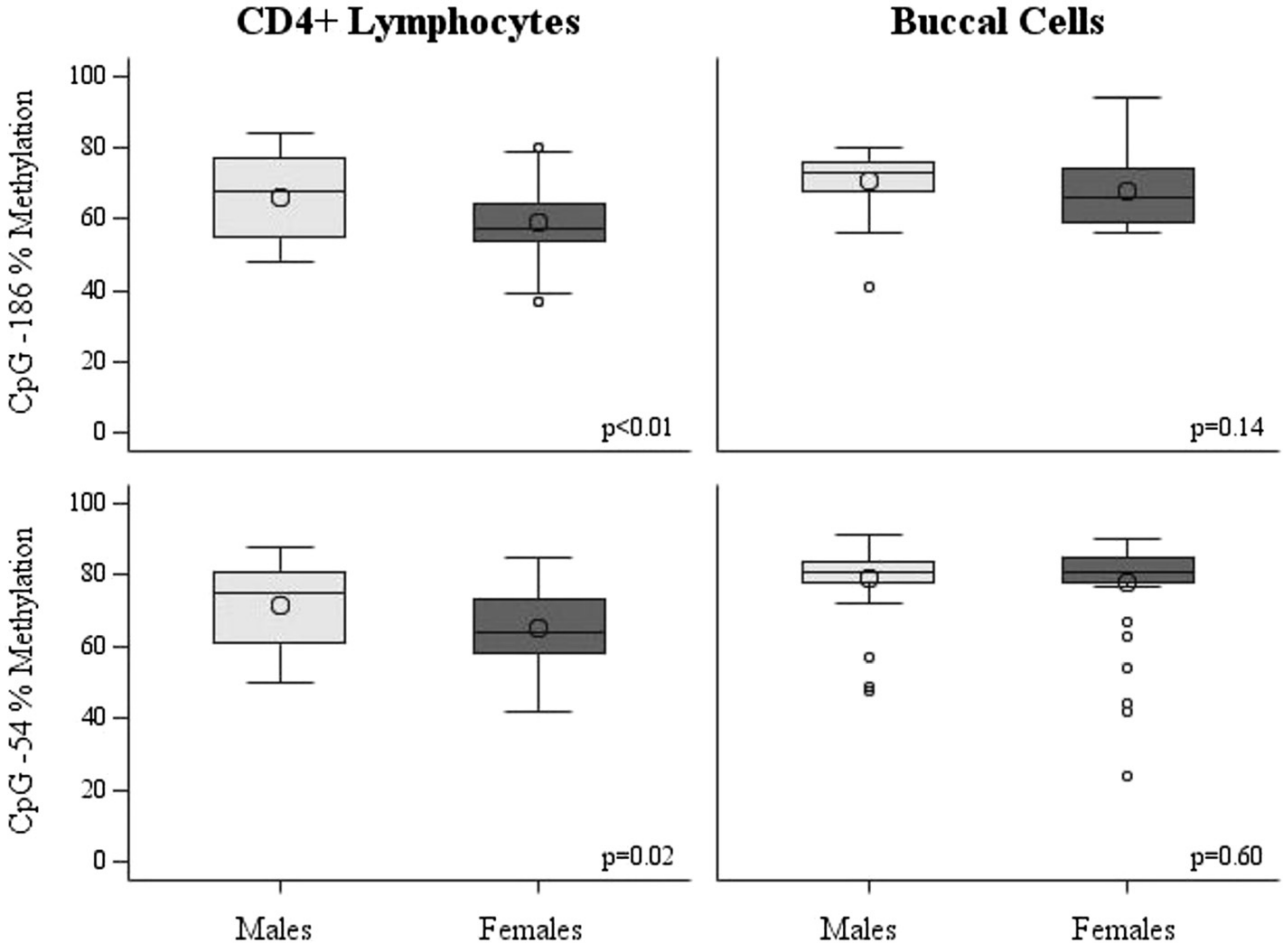
**Comparison of IFNγ promoter methylation levels by tissue type and CpG site stratified by sex.** n = 31 males, n = 43 females. Males had significantly higher CD4+ methylation levels at both CpG sites (-186: *P* < 0.01, -54: *P* = 0.02), however, sex-specific differences were not identified in buccal cells (-186: *P* = 0.14, -54: *P* = 0.60).

In order to identify age- and sex-related patterns of IFNγ promoter methylation in a cohort of allergic asthmatics CD4+ lymphocytes and buccal cells (matched pairs) were collected from 27 children and 47 adults. A total of 60% of the adults were diagnosed with asthma in childhood by the age of 12 years. The methylation of CD4+ lymphocytes was higher in children compared with adults at both CpG -186 (*P* <0.01) and CpG -54 (*P* <0.01). The methylation of buccal cell DNA was also higher in children compared with adults at CpG -186 (*P* = 0.03) but not at CpG -54 (*P* = 0.66) (Figure 2). The methylation of CD4+ lymphocytes was higher in males compared with females at both CpG -186 (*P* <0.01) and CpG -54 (*P* = 0.02), however there was no significant sex-related difference in buccal cell methylation (CpG -186: *P* = 0.14, CpG -54: *P* = 0.60) (Figure 3).

In a multivariable regression model both age and sex were significant predictors of IFNγ CpG -186 methylation (*P* <0.01) in CD4+ lymphocytes. Sex (n = 31 males, n = 43 females) was a significant modifier of the effect of age on methylation in CpG -186. As age in males increased by one year absolute CD4+ lymphocyte methylation decreased by 0.52%, whereas as age in females increased by one year absolute methylation decreased by 0.28%. Age was also a significant predictor of methylation in CpG -54, although there was no significant interaction by sex.

### Comparison of methylation levels between CD4+ lymphocyte and buccal cells

**Figure 4:**
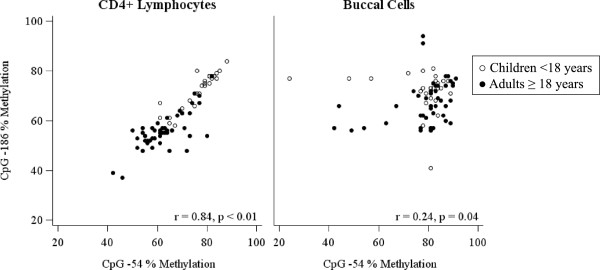
**Relationship between IFNγ promoter methylation at neighboring CpG sites (-186 and -54) stratified by cell type.** n = 27 children (clear dots) and n = 47 adults (filled dots). The methylation level was highly correlated between neighboring CpG sites in CD4+ lymphocytes (r = 0.84, *P* <0.01) and weakly correlated across neighboring CpG sites in buccal cells (r = 0.24, *P* = 0.04).

As DNA methylation in different tissue types can vary and appropriate biomarkers for epigenetic asthma research are needed, we sought to determine the relationship between CD4+ lymphocyte and buccal cell IFNγ promoter methylation in allergic asthmatics. The methylation level was lower in CD4+ lymphocytes compared with buccal cells at both IFNγ promoter CpG -186 (*P* <0.01) and CpG -54 (*P* <0.01). Significant correlation was observed between CD4+ lymphocyte and buccal cell methylation at CpG -186 (r = 0.24, *P* = 0.04) (Figure 4). However, there was no correlation in methylation between the differing cell types at CpG -54 (r = -0.03, *P* = 0.78). When the data were stratified by age group, correlations were not detected at CpG -186 (children: r = 0.19, *P* = 0.34; adults: r = -0.15, *P* = 0.31) nor CpG -54 (children: r = 0.16, *P* = 0.42; adults: r = -0.04, *P* = 0.77). When the data were stratified by sex, correlation was detected between differing cell types in males at CpG -186 (r = 0.42, *P* = -0.02) but not CpG -54 (r = 0.04, *P* = 0.84). No correlation was detected between differing cells in females (CpG -186: r = 0.06, *P* = 0.69; CpG -54: r = -0.1, *P* = 0.54).

### Comparison of methylation levels between CpG sites by cell type

In order to explore relationships between neighboring CpG sites in different tissue types we compared methylation of the two IFNγ promoter CpG sites in CD4+ lymphocytes and buccal cells. DNA methylation correlated highly between CpG -186 and -54 in CD4+ lymphocytes (r = 0.84, *P* <0.01), but weakly in buccal cells (r = 0.24, *P* = 0.04). When stratified by age group, the strong correlation between CpG sites in CD4+ lymphocytes remained in both strata (children: r = 0.88, *P* <0.01; adults: r = 0.63, *P* <0.01). A significant correlation emerged between CpG sites in adult buccal cells (r = 0.44, *P* <0.01), but not in child buccal cells (r = -0.14, *P* = 0.49).

### Comparison of methylation and gene expression

DNA methylation in the promoter region of IFNγ has been negatively correlated with RNA gene expression *in vitro*[16] and *in vivo*[31]. Thus we sought to validate this finding in our cohort of allergic asthmatics. RNA was analyzed for gene expression in 24 children and 44 adults (missing data n = 6) Relative IFNγ gene expression, normalized to glyceraldehyde 3-phosphate dehydrogenase (GAPDH) gene expression using the 2^ΔCt^ method, negatively correlated with CD4+ lymphocyte methylation at CpG -186 (r = -0.38, *P* <0.01) and CpG -54 (r = -0.28, *P* = 0.02).

## Discussion

We have demonstrated that in a cohort of children and adults with allergic asthma, age and sex are important predictors of IFNγ promoter DNA methylation. IFNγ promoter methylation was higher in the CD4+ lymphocytes and buccal cells of children compared with adults, and higher in males compared with females in CD4+ lymphocytes but not buccal cells. IFNγ promoter methylation also behaved differently between tissue types. Methylation levels were lower in CD4+ lymphocytes compared to buccal cells, methylation between the two tissue types did not correlate, and neighboring CpG methylation correlated in CD4+ lymphocytes but not in buccal cells. These findings underscore the importance of age- and sex-related differences in IFNγ promoter methylation, a phenomenon that likely also exists in other asthma regulatory genes but is understudied. In addition, our findings highlight the significance of varying methylation patterns in different tissue types, and we present an example of the concept that neighboring CpG sites behave differently in differing tissues. This knowledge is integral in developing appropriate biomarkers for epigenetic asthma research.

Asthma prevalence is known to vary by age and sex such that in early childhood males are more likely to have asthma symptoms than females, while this relationship seems to reverse in adolescents resulting in more female adult asthma [25,26]. In our study population 60% of the adults were diagnosed with asthma in childhood, therefore the differences we observed are unlikely to be due to phenotypic differences related to childhood versus adult onset asthma. In a cohort of asthmatics and non-asthmatics, Naumova *et al.*[23] reported lower mean methylation levels in males compared with females, as well as slight increases in methylation with age in the zona pellucida binding protein 2 (ZPBP2) promoter, a gene found in the 17q12-q21 chromosome region that has been linked to childhood onset of asthma. Although the direction of the sex- and age-specific methylation differences in ZPBP2 is opposite to that of IFNγ, both our and the Naumova studies highlight the significance of age and sex in DNA methylation that may modify genetic effects in diseases like asthma. It is plausible that the epidemiologic variation in asthma prevalence may be attributable to varying DNA methylation patterns with sex and age. Support for the former case can be derived from prior studies in rats and mice that showed estrogen hormone administration altered DNA methylation and the gene expression of multiple genes in several organs [32,33]. Therefore it can be postulated that hormone-related changes with puberty may alter DNA methylation and histone modification patterns in asthma genes like IFNγ, thus increasing or decreasing the risk of diseases like asthma [34]. In this current study design we did not have power to assess sex-related methylation differences in child versus adult age groups, however, this is an important area for future research.

Biomarkers from both systemic (IgE, periostin, and circulating eosinophils) and airway (fractional exhaled nitric oxide (FeNO) and sputum eosinophils) sources are used in asthma research, especially among those with the allergic phenotype. However it is unclear which is superior in terms of accessibility and prediction of disease progression. Therefore in developing novel epigenetic biomarkers in asthma, it is critical that we distinguish key epigenetic differences in systemic versus airway tissues. Similar to Stefanowicz *et al.* who identified differential patterns of methylation in PBMCs and airway epithelial cells by microarray [27], using a different technique we too found methylation varied between systemic cells, CD4+ lymphocytes, and airway surrogates (buccal cells). Our approach was to focus on the allergic asthma counter-regulatory IFNγ gene promoter, susceptible to epigenetic regulation following environmental exposures [16], and due to the more sensitive approach of pyrosequencing we identified cell/tissue specific methylation differences in a gene not previously reported by Stefanowicz *et al.* The strong correlations between CpGs -186 and -54 in CD4+ lymphocytes and weaker correlations between the same sites in buccal cells reported here also highlight the varying behavior of neighboring CpG methylation in different tissues. We sampled buccal cells as they are an easily accessible population of cells that have been proposed as an appropriate surrogate for lower airway epithelial cells. A strong correlation in IFNγ gene expression has been demonstrated between buccal and lower airway epithelial cells in adult smokers [30]. In addition, buccal cell methylation levels in children in a different asthma pathway (arginase 1 and 2 and inducible nitric oxide synthase (iNOS)) have been associated with the airway inflammatory marker FeNO [35-37], suggesting DNA methylation in buccal cells may be clinically relevant to airway disease.

Previously, Jones and Chen suggested that because of its rapid methylation upon stimulation, the more proximal CpG -53 site, compared with the five other murine IFNγ promoter CpG sites they examined, is the key CpG involved in the suppression of IFNγ transcription [12]. Gonsky *et al.*[10] replicated these findings in subjects with inflammatory bowel disease and similarly demonstrated methylation of the corresponding human IFNγ CpG site -54 significantly inhibited gene expression in PBMCs and intestinal lamina propria cells. In our allergic asthma cohort CpG -186 emerged as an interesting site based on the significant correlations in methylation between CD4+ lymphocytes and buccal cells at this CpG and not -54. In addition, CpG -186 methylation was predicted by both age and sex, whereas -54 was only significantly predicted by age and not sex in multivariable regression models. These neighboring CpG sites appear to behave differently in the different tissues examined given the correlation between the two sites in CD4+ lymphocytes but not in buccal cells. These findings suggest there may be a unique methylation signature that varies by tissue type offering different information that needs to be further related back to disease phenotypes.

The link between IFNγ promoter DNA methylation and gene expression has been explored in several *in vitro* studies [12,13]. *In vivo*, Kohli *et al.* described significant negative correlations (r = -0.75) between IFNγ DNA methylation and the gene expression of T effector cells of 7 to 18-year-old non-asthmatics [31]. In contrast to the high inverse correlation in the aforementioned study, we found significant but weak inverse correlation between IFNγ DNA methylation and gene expression in the CD4+ lymphocytes of adults and children with asthma suggesting that promoter methylation is not the only driver of IFNγ gene expression. In a study of IFNγ methylation in cord blood mononuclear cells and adult peripheral blood mononuclear cells, White *et al.* described significant methylation in non-CpG sites (CpA and CpT methylation) and hypothesized methylation at these sites may enhance CpG methylation suppression of gene activity [22]. In addition, there is an emerging body of literature on 5-hydroxymethylation [38] as well as on the significant role of RNA polymerase in gene transcription, emphasizing the complexity of the epigenome and indicating a more complex relationship with downstream transcriptional activities.

We acknowledge that by sampling a select group of asthmatics that were both exposed and sensitized to indoor allergens, these findings may not be generalizable to the population although, our goal was to understand a key allergic regulatory gene in an allergic patient population. IFNγ promoter methylation needs to be compared between asthmatics and healthy controls in future studies to further validate the importance of this region, to determine if the observed patterns are the result of the disease state alone, and to confirm the significant age- and sex-related differences we describe. Another limitation of our study is that we compared DNA methylation in buccal cells (a mixed-cell population) to CD4+ lymphocytes (a very select population). In our study methylation across CpG sites was more consistent and correlated in CD4+ lymphocytes compared with buccal cells, suggesting methylation of the systemic CD4+ lymphocytes is more consistent across the gene region than in the local buccal cells. However, further studies linking methylation in varied cell types to asthma outcomes are needed to determine the superiority of CD4+ lymphocytes versus buccal cells as a biomarker for allergic asthma.

## Conclusions

Our findings highlight significant age-, sex- and tissue-related differences in IFNγ promoter methylation in allergic asthmatics. These associations are likely present and need to be investigated further in other asthma-related genes. This knowledge is critical to our understanding of methylation in the allergic asthma pathway and in the development of appropriate biomarkers for future epigenetic asthma studies.

## Methods

### Study population

Subjects were recruited for the randomized control trial ‘Comparative effectiveness of environmental intervention and standard care in ability to reduce pharmacologic therapy for asthma’ (American Recovery and Reinvestment Act (ARRA): R01 HS019384). Inclusion criteria were as follows: clinical diagnosis of asthma, use of controller medication, reversibility of airway obstruction on spirometry post-bronchodilator or positive methacholine challenge, positive skin prick test or specific IgE test to common indoor allergens (dog, cat, cockroach, dust mite, mouse), and elevated allergen in the home dust sample. Baseline buccal and blood samples were collected prior to randomization to control or intervention groups. All subjects in which both buccal and blood samples were collected were included in the analysis (27 children and 47 adults). Informed consent and assent were obtained from all participants prior to their participation in the study. This study was approved by the Columbia University Institutional Review Board.

### Collection of buccal cell samples

Buccal cell samples were collected using the CytoSoft cytology brush (Fischer Scientific, Pittsburgh, Pennsylvania, United States), as published by our group [17]. Each participant rinsed his or her mouth with water and then brushed the inside of his or her cheeks for one minute. The swab was then immediately placed into 600 μl of cell lysis solution (Qiagen Sciences, Germantown, Maryland, United States). This process was repeated five times for each participant yielding two buccal samples each for DNA analysis.

### Collection and isolation of CD4+ lymphocytes

PBMCs were isolated from whole blood samples using the Ficoll-Paque (GE Healthcare, Uppsala, Sweden) method. CD4+ lymphocytes were then isolated using CD4 + MicroBeads according to the MACS® Miltenyi Biotec (Auburn, California, United States) protocol.

### DNA extraction, quantification, and bisulfite conversion

Buccal cell and CD4+ lymphocyte DNA extractions were performed using Puregene buccal cell core kits (Qiagen Sciences, Germantown, Maryland, United States) according to the manufacturer’s instructions, except all centrifugations were conducted at 4°C instead of room temperature. The two buccal samples obtained from each participant were combined after individual extraction. Extracted DNA was quantified using a NanoDrop spectrophotometer (Thermo Scientific, Wilmington, Delaware, United States). Bisulfite conversion was performed on 200 ng of genomic buccal cell and CD4+ lymphocyte DNA using Zymo Research’s EZ DNA methylation kit (Irvine, California, United States) and the manufacturer’s instructions. Samples were incubated under the alternative incubation conditions for Illumina Infinium methylation assay (San Diego, California, United States) with an increased number of cycles (20 cycles of 95°C for 30 seconds and 50°C for 15 minutes) [17,39].

### PCR amplification and pyrosequencing

The primers for performing PCR and the PCR product sequencing (Table 1) were designed using PyroMark Assay Design 2.0 software (Qiagen, Valencia, California, United States) for the regions of interest: IFNγ CpG -54 and -186. These targeted areas were chosen based on previous studies from our group and others that demonstrate change in methylation associated with allergic outcomes and asthma diagnosis [5,14,15,31]. PCR reactions were performed with Qiagen Hot Star Taq DNA polymerase reagents (Qiagen Sciences, Germantown, Maryland, United States) with the following concentrations for each ingredient in the PCR mixtures: 1 × PCR buffer, 0.5 μM deoxynucleotide triphosphates (dNTP), 0.5 μM forward primer, 0.5 μM reverse primer and parameters highlighted in Table 1. The PCR product was sequenced using a PyroMark Q24 pyrosequencer after verifying the positive PCR products by visualizing the appropriately sized band on a 1.2% agarose gel. EpiTect high and low methylated control DNA (Qiagen Sciences, Germantown, Maryland, United States) were included with every pyrosequencing experiment. Batches were repeated if high methylation control was <85% methylated (mean 93.5%, SD 4.3) and low methylation control was >10% methylated (mean 3.8%, SD 1.8). Replicate pyrosequencing results for n = 11 matched CD4+ lymphocyte and buccal samples yielded good concordance correlation (CD4+ cells r_c_ = 0.93, buccal cells r_c_ = 0.94).

**Table 1:** Primers and amplification conditions for PCR, pyrosequencing, and RT-qPCR experiments

**Gene/region**	**Assay**	**Primers**	**PCR Conditions**
IFNγ promoter CpG -186	PCR and Pyrosequencing	F: 5’-biotin-AGATGGTGATAGATAGGTAGGGATGATA-3’	95°C, 15 min; 45 cycles of 95°C, 30 sec; 55°C, 30 sec; 72°C, 30 sec; 72°C, 10 min; 4°C hold
		R: 5’-TCCCACCAAAATAACACAAATAAACAT -3’	
		S: 5’-AAATAAACATAATAAATCTATCTCA-3’	
IFNγ promoter CpG -54		F: 5’ATGTGTTGTATTTTTTTTGGTTGTTGGTAT-3’	
		R: 5’-biotin-TATCATCCCTACCTATCTATCACCATCTC-3’	
		S: 5’-ATTGAAGTTTTTTGAGGATT-3’	
IFNγ promoter (target)	RT-qPCR	F: 5’-TCGGTAACTGACTTGAATGTCCA-3’	95°C, 3 min; 40 cycles of 90°C, 10 sec; 55°C, 30 sec; 4°C hold
		R: 5’-TCGCTTCCCTGTTTTAGCTGC-3’	
GAPDH (internal control)		F: 5’-ACAACTTTGGTATCGTGGAAGG-3’	
		R: 5’-GCCATCACGCCACAGTTTC-3’	

F (forward primer), R (reverse primer), S (sequencing primer), IFNγ (interferon gamma), GAPDH (glyceraldehyde 3-phosphate dehydrogenase), min (minutes), sec (seconds).

### RNA extraction and reverse transcription

RNA was extracted from isolated CD4+ lymphocytes and stored in TriReagent (Molecular Research Center, Cincinnati, Ohio, United States) according to the manufacturer’s instruction with 0.2 ml of chloroform and the following exceptions: 0.6 ml of isopropanol was used for RNA precipitation, 1 ml of 70% ethanol was used to wash RNA, and all centrifugations were performed at 4°C instead of room temperature. RNA was quantified using a NanoDrop spectrophotometer (Thermo Scientific, Wilmington, Delaware, United States). cDNA synthesis was performed using the SuperScript First-Strand Synthesis System (Life Technologies, Grand Island, New York, United States) according to the manufacturer’s instructions.

### Real-time quantitative PCR (RT-qPCR)

RT-qPCR was performed using iTaq Universal SYBR Green Supermix (BioRad, Hercules, California, United States) with up to 200 ng of cDNA and 1 μl of primer pair mix on a BioRad real-time PCR system. Expression of IFNγ (RefSeq: NM_000619) was normalized to that of glyceraldehyde-3-phospate dehydrogenase (GAPDH) (RefSeq: NM_001256799). The primers and conditions used for amplification are noted in Table 1. Procedures were performed twice per sample . Results were calculated using the 2^ΔCt^ relative to the control gene, GAPDH.

### Statistical analysis

Spearman’s correlations were performed for the data that were not normally distributed and Pearson’s correlations were performed for all others. All correlations, student’s t-tests, and linear regression models were performed using SAS 9.4 software (Cary, North Carolina, United States). In order to determine effect modification of age by sex a cross product term of these two variables was included in the multivariable linear regression model. We adjusted for tobacco smoke exposure and asthma medication use, which were not significant predictors nor did they influence our main effects, therefore we eliminated these variables in our final model.

## Abbreviations

AEC: Airway epithelial cells; CpG: Cytosine phosphate guanine; dNTP: deoxynucleotide triphosphates; FeNO: Fractional exhaled nitric oxide; GADPH: Glyceraldehyde-3-phosphate dehydrogenase; IFNγ: Interferon gamma; IGF2: Insulin growth factor 2; iNOS: Inducible nitric oxide synthase; OVA: Ovalbumin; PAH: Polycyclic aromatic hydrocarbon; PBMC: Peripheral blood mononuclear cells; PCR: Polymerase chain reaction; RT-qPCR: Real time qualitative polymerase chain reaction; Th: T helper; Treg: T regulatory; ZPBP2: Zona pellucida binding protein 2.

## Competing interests

The authors declare that they have no competing interests.

## Authors’ contributions

SLD participated in the design, conduction and coordination of the study, its statistical analysis and drafted the manuscript. RR participated in the conduction and coordination of the study. DT participated in the design of the study and edited the manuscript. CM participated in conduction of the study and edited the manuscript. SN and MS participated in conduction and coordination of the study. DM facilitated data management and participated in statistical analysis. MK and ED participated in the design of the study and edited the manuscript. RLM conceived and designed the study and helped draft and edited the manuscript. All authors read and approved the final manuscript.
